# Development of digital learning tools for medical education with agile scrum methodology

**DOI:** 10.1186/s12909-025-08270-9

**Published:** 2025-12-15

**Authors:** Jeffrey S. Puncher, Sathya Karunananthan, Selya Amrani, Douglas Archibald, Nicole Brunet, Sylvie Forgues-Martel, Alexander Hajjar, Mary Helmer-Smith, Kheira Jolin-Dahel, Tess McCutcheon, Emily Seale, Claire Sethuram, Lina Shoppoff, Sara Trincoa-Batra, Parisa Rezaiefar, Marisa Duval, Ahmed Husseini Orabi, Clare Liddy

**Affiliations:** 1 Department of Family Medicine, University of Ottawa, Ottawa, Canada; 2 C.T. Lamont Primary Health Care Research Centre, Bruyère Health Research Institute, Ottawa, Canada; 3 Faculty of Health Sciences, University of Ottawa, Ottawa, Canada; 4 Faculty of Medicine, University of Ottawa, Ottawa, Canada; 5 Faculty of Medicine, Queen’s University, Kingston, Canada; 6 Ontario eConsult Centre of Excellence, The Ottawa Hospital, Ottawa, Canada

**Keywords:** Digital learning tools, Agile, Scrum, Medical education, Digital health

## Abstract

**Background:**

The COVID-19 pandemic has underscored the importance of digital learning tools in medical education. However, there is little evidence exploring how or whether such tools are being developed for family medicine curricula. We provide a narrative analysis of how the University of Ottawa’s Department of Family Medicine (DFM) developed innovative learning tools using an interdisciplinary, research-based approach and Agile Scrum Methodology.

**Methods:**

In March 2020, the DFM created an interdisciplinary team to support development of digital tools for medical education. Members of the DFM were invited to participate in the project during two faculty-wide webinars held on May 13, 2020. Participants identified three topic areas for which digital learning tools were to be created: Choosing Wisely Canada recommendations, Hypertension, and Quality Improvement (QI). Representatives from the Faculty of Engineering were recruited to support IT development for the tools, while researchers from the Bruyère Research Institute provided support for methodology and analysis.

**Results:**

Three teams developed prototypes for digital learning tools: a “choose your own adventure” game to teach Choosing Wisely Canada criteria, an interactive hypertension clinic, and a “QI escape room” focused on quality improvement strategies. One team created a website to host learning tools, and the final team generated an evidence library for product development.

**Conclusion:**

Adhering to these methodologies helped us to manage interdisciplinary teams and support their success, with all five teams completing their objectives. The interdisciplinary and incremental approach of Scrum methodology allowed for gaps to be identified and addressed in real time. Scrum demonstrates promise and should receive further consideration as a method for developing learning tools in medical education.

## Background

The COVID-19 pandemic has underscored the importance of digital learning tools in medical education [1]. Digital learning tools refer to any computer-based resource, mobile application, electronic game or resource that supports, enhances, or contributes to education [2, 3]. Though the value of digital learning tools is well documented for learning outcomes and professional development [3–9], there is limited evidence to guide the identification and development of these resources for integration into family medicine curricula. We recognized the need to address this issue by establishing an interdisciplinary team and applying efficient methods to create effective digital learning tools for medical trainees.

In response to the significant changes to traditional summer learning opportunities imposed during the pandemic, the Department of Family Medicine (DFM) at the University of Ottawa recruited a team of medical and engineering students, faculty, staff, and researchers to develop digital learning tools for medical education. A key first step to this process involved choosing a methodology to guide tool development, as research has shown that a poor foundational framework inhibits the success of such tools in medical education [10]. Interdisciplinary methods are the gold standard for developing digital tools for clinical settings to ensure the tool is both useful and usable [11], so our team included participants outside of the DFM and explored potential approaches outside of the discipline as well. We selected Agile Scrum Methodology, an innovative practice that has been used in several disciplines, including business and software development.

Agile software development is guided by four key priorities: valuing individuals and interactions, prioritizing working software over documentation, collaborating with customers, and responding to change rather than following a plan [12]. Scrum is an Agile project management methodology that provides a framework for teams to structure their work based on Agile principles and philosophy [13]. To our knowledge, Agile Scrum Methodology has not previously been applied in an academic medicine setting.

The purpose of this paper is to provide a narrative analysis of how our department developed digital learning tools for medical education using Agile Scrum Methodology. By discussing our process and outcomes, we hope to encourage other institutions and clinicians to become involved in developing serious games and provide a useful example for how this can be done effectively by involving students in the process. Our experience illustrates that Agile Scrum Methodology can be an efficient and novel approach to resource development in medical education.

## Methods

### Project conception

COVID-19 reached Canada in early 2020, and, by March of that year, the emerging pandemic caused mass shutdowns of on-site learning among Canada’s universities and greatly restricted clinical instruction. As a result, many students at the University lost the placements they had obtained for the summer. Likewise, the inability for students to attend classes emphasized the importance of developing digital learning tools, which have proven effective in many instances [2, 3, 14]. They are especially useful in a pandemic or other situations where in-person learning is restricted.

Seeing an opportunity among these challenges, the DFM reached out to the Faculty of Engineering, as many of their students had their planned co-op placements cancelled for the summer due to the pandemic. The DFM connected interested medical students with software engineering students, recruiting volunteers to develop evidence-based digital tools that would support medical education. Members of the DFM were invited to participate in the project during two faculty-wide webinars held on May 13, 2020. Team members identified three topic areas for which digital learning tools were to be created: Choosing Wisely Canada Recommendations [15], Hypertension, and Quality Improvement. To support evaluation of the process, the DFM invited participants from the Bruyère Research Institute. Following the department webinar, a webinar series was arranged to inform interested students and researchers of the projects and form the project teams. In May 2021, the project was expanded to develop additional digital learning tools.

### Agile scrum methodology

We employed Agile Scrum Methodology to facilitate/guide product development. Agile Scrum Methodology is an iterative process that allows for dynamic collaboration and evaluation during product development [13, 16]. The process requires a collaborative team with diverse skillsets and backgrounds, who remain engaged throughout the project and work together to complete all features of the project. This approach allows for effective and timely development of digital learning tools while providing a unique collaborative and team-based experience for students [17, 18].

A Scrum breaks down projects into smaller tasks to be completed incrementally by the project team, allowing them to track progress, replan, and reprioritize incrementally and as needed [16, 18]. During a Scrum, the project team members remain constant throughout the project, thus facilitating collective ownership, effective knowledge exchange, and shared responsibility [16]. Scrums consist of various events that typically take place within a two-to-four-week increment called a Sprint. During a Sprint, each Scrum Team completes prioritized features of the project according to established prioritization frameworks like MoSCoW and RICE scoring [19]. These features must meet the acceptance criteria specific to each user story and adhere to the overarching definition of “Done” created by the project team, ensuring they are of high quality and ready for release. Each day, teams meet individually for a fifteen-minute daily Scrum to review the progress of their work. Daily check-ins allow the larger group to tackle problems quickly, reducing the time spent waiting for feedback, reducing technical debt, and creating more opportunities to allocate resources where needed.

“Sprint Reviews” are held at the conclusion of each Sprint. At the Sprint Review, the teams present the work completed during the Sprint that met the definition of Done. The stakeholders evaluate the Sprint deliverables and provide feedback. During the “Sprint Retrospective”, the scrum teams share lessons learned, and opportunities for improvement, identifying what worked well during the Sprint and what could be improved upon for the next Sprint. Sprint Reviews and Retrospectives are followed by “Sprint Planning”, in which teams collaborate to identify goals, decide which “Product Backlog” items to include, and plan how to complete them. The team creates a “Sprint Backlog”, which is a detailed plan that is aligned with priorities for the next Sprint and divides tasks among team members.

### Teams

Five Scrum Teams were created: three to develop a digital tool for the identified content areas (Quality Improvement, Hypertension, and Choosing Wisely Canada Recommendations [15]), one to review the existing literature about digital learning tools and collect information to help inform digital tool development and a program of research, and one to develop a website to house the tools. Each Scrum Team had six to eight members. Team members consisted of medical students, software engineering students, DFM faculty members, DFM staff, and researchers.

## Results

**Fig. 1:**
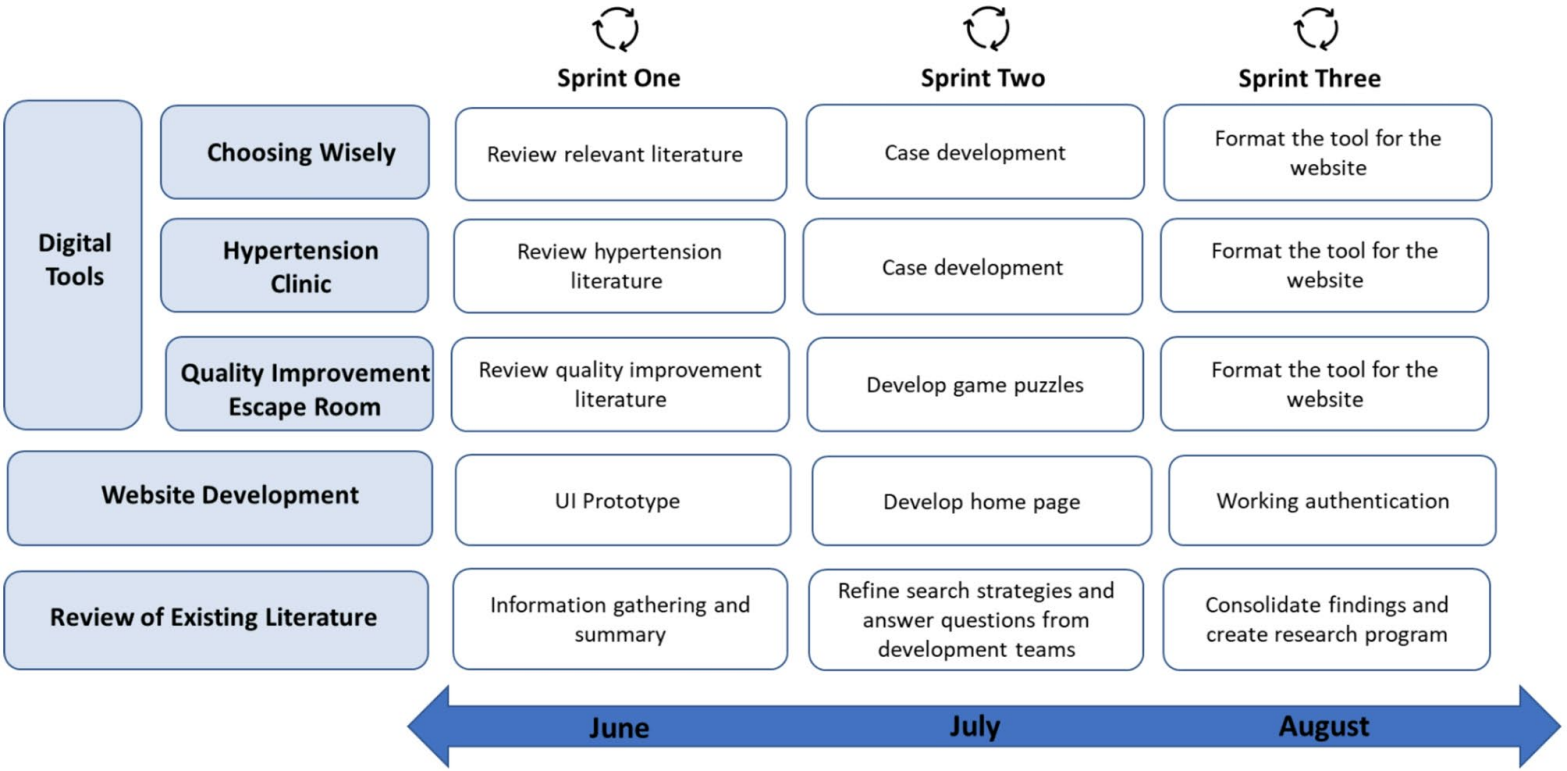
Project Sprints

Over the course of the project, each Scrum Team completed three Sprints **(**Fig. 1). The outcomes for each Scrum Team are outlined below.

### Digital tool development teams

Over the three-month Scrum period, three of the five teams focused on developing a digital learning tool for their assigned topic area. The educational objectives of these tools were to: (1) familiarize family medicine clerkship students with the Choosing Wisely Canada recommendations; (2) teach medical students the Hypertension Canada Guidelines; and (3) teach first-year family medicine residents basic quality improvement knowledge.

#### Choosing Wisely

This tool is a “Choose Your Own Adventure” game based on the Choosing Wisely Canada recommendations for Family Medicine [15]. The tool aligns with five roles in the CANMEDS-Family Medicine competency framework (Family Medicine Expert, Communicator, Leader, Scholar, and Professional) and targets six of the Core Professional Activities defined by the College of Family Physicians of Canada (CPA 1, CPA 2, CPA 8, CPA 12, CPA 22 and CPA 31)[20]. The objective of the tool is to help students completing clerkships in family medicine to determine the course of action for a patient presenting with a specific issue. In this game, players are asked to work through hypothetical cases, choosing from a list of recommendations and earning points for correct answers. In Sprint 1, team members conducted a review of the relevant literature to identify key aspects of the Choosing Wisely Canada criteria. These items were used to inform patient cases developed in Sprint 2. During Sprint 3, team members formatted the tool for uploading onto the website. At the end of the Scrum, ten patient cases had been developed, and the engineering students had designed the game platform. Next steps included developing additional cases and completing the online launch of the tool. We started this in 2022 and went live in both official languages for family medicine students in 2025. We found that the development time was significantly prolonged by our choice to use part-time student developers who worked with us for cycles of 3–4 months. However, this strategy was financially efficient and provided a valuable learning experience for the students.

#### Hypertension Clinic

**Fig. 2:**
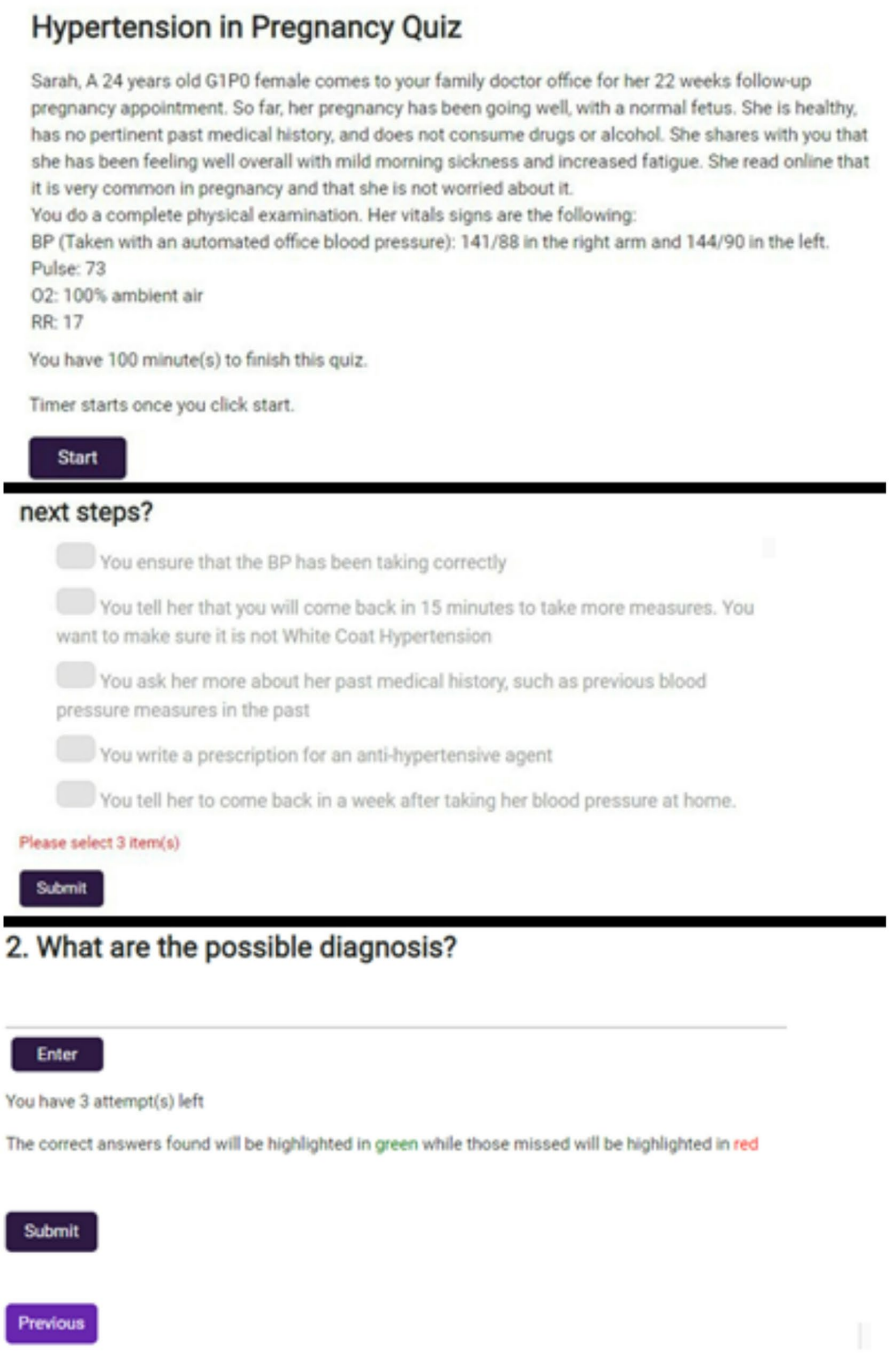
Screenshots from Hypertension Quiz prototype

The second tool, “Hypertension Clinic,” is an interactive game designed to help medical students at all levels learn Hypertension Canada Guidelines [21] as they apply to different healthcare settings, including hospital wards, family medicine clinics, and emergency rooms. The game features short interactive patient-provider cases based on the 2020 Hypertension Canada Guidelines (Fig. 2). Players have the opportunity to work through different cases, earning points for correct answers and losing points for incorrect answers or for taking too much time.

Team members conducted a review of existing materials for Sprint 1, including hypertension literature and previous self-learning modules. In Sprint 2, team members used this information to develop eleven potential scenarios. Team members each worked on a scenario individually, then presented it to the team for critique and refinement, which included review by a family physician and family medicine resident. After applying the feedback, team members then started on a new case, with each member developing 3–4 cases during the Sprint. For Sprint 3, the group formatted the game for the website. The scenarios were further adjusted and formatted for the online platform by the software engineers while maintaining the integrity of the clinical content. Eleven scenarios were completed during the Scrum. Next steps included finalizing the remaining cases and launching them online, which was started in 2022 but has not been completed due to human resource limitations.

#### Quality Improvement

The aim of this tool was to teach first-year family medicine residents basic quality improvement (QI) knowledge [22], and help them build teamwork, collaboration, and communication skills. To this end, team members developed a “QI Escape Room,” in which players work together in a virtual reality escape room setting to solve puzzles based on QI knowledge and complete the case.

Team members reviewed the relevant literature in Sprint 1, which they used to develop puzzles in Sprint 2. They formatted the tool for uploading to the website in Sprint 3. At the end of the Scrum, the team had created final drafts of all the puzzles and had written additional clues to support learners who get stuck. Next steps included final programming of the game, pilot testing among family medicine residents, adding multi-player functionality, and optimizing for mobile devices and virtual reality. This process was started in 2021 and completed in 2022. In addition to student developers, we used a $90,000 eCampus grant to work with a professional software development company. This allowed us to meet the required timeline while continuing to involve students. This application is now integrated into our family medicine curriculum and is used by our residents each academic year as part of their QI training.

### Review of existing literature team

This team consisted of six students, four researchers, and three DFM staff, who worked together to explore existing literature about approaches to digital medical education, search for relevant information for the product development teams, and identify gaps in the literature.

Beginning in Sprint 1, the team gathered peer-reviewed articles and grey literature focused on digital learning tools for medical education into a Zotero library. Eligible items included publications about concept development, applied content, virtual reality, research design in game-based learning, and technical requirements. The three teams working on digital tool development were able to draw on this library to support their work and could submit questions related to their projects to the literature review team, who would research them and provide relevant material. On the occasions where they were unable to find published or reference material to answer questions from one of the development teams within the Sprint’s time period, these queries were identified as knowledge gaps and set aside for additional exploration in the future.

By the end of Sprint 3, the team had completed a review of 89 unique digital learning resources for medical education and compiled a glossary defining 33 acronyms and 46 common terms from the literature. They also identified several future research priorities, which include conducting a needs assessment survey of medical students and family medicine residents, and systematically reviewing the literature on key education delivery and design considerations for digital tools in medical education.

### Website development team

The website development team created a unique platform to host the digital learning tools called the Innovation Portal [20]. They began by gathering requirements from product owners and researching available platforms. While web technologies that met most of the criteria existed, it was clear that to satisfy certain requirements, such as the ability to share the tools publicly and track usage, a custom platform would need to be developed. The website development team was integrated into each product development team to design the technical aspects of the products and integrate these directly into a unique section on the website.

### Expansion

After the successful completion of the initial project above, the Scrum method was used to develop additional digital learning tools. All tools, including the three developed in the initial Scrum, are available through the DFM Innovation Portal [20]. The portal currently hosts three virtual reality (VR) experiences, seven e-learning modules, and seven experimental outreach modules. The VR experiences include the QI Escape Room described above, a Scuba VR application that helps learners understand the causes and treatments of stress and anxiety during a simulated dive, and a Contamination Control & Spread VR that uses a virtual playthrough of walking through a school to illustrate how germs can be transmitted and how their spread can be controlled. The e-learning modules include an Anti-Racism Curriculum, the Gynecologic Procedures for Primary Care (GP4PC) project, Long-Term Care, The Musculoskeletal (MSK) Teaching Module, and The Veterans Health e-Learning Modules, in addition to the Choosing Wisely and Hypertension Clinic modules that were initially developed. The educational competencies targeted by each tool are listed on its page in the portal, where possible [20]. The experimental outreach program aims to augment the output of the DFM and innovate in new areas by building stronger, multidisciplinary links across the University of Ottawa community, including current University of Ottawa students and Family Medicine professionals. Its current projects include Doc in a Box, Mental Health Connections, My Medical Records Application, SIM City Ottawa, Unattached Patient, UG Exam Database, and Resident Intervention.

### Application

Residents used the Anti-Racism Curriculum for the first time in the 2023–2024 academic year, with 150 DFM residents completing the modules to date. Each year, new PGY1s (residents in their first year of formal postgraduate training) will be instructed to complete the online modules and then participate in an in-person discussion of the content. Several other programs at the University of Ottawa have also requested access to the Anti-Racism Curriculum content. The VR escape room was introduced in the same academic year and 120 learners to date have used it as part of their QI training, with an additional 84 expected to participate in November 2025. The Scuba VR program has recently been piloted with first responders and emergency healthcare workers in Alberta and Ontario for the self-management of post-traumatic stress injuries. Preliminary feedback from participants indicated that they enjoyed the VR activity and found it to be a positive experience.

## Discussion

While Agile Scrum Methodology is a well-established framework in other fields like business and software development, its application to developing digital tools for medical education was innovative for our department. This approach was highly effective, as all five teams completed the cycle and met the initial project objectives: the Digital Tool Development Teams successfully created prototypes of the tools, the Website Development Team created a custom website to house the different tools, and the Literature Review Team synthesized current evidence to compile a list of digital tools for medical education, a glossary, and research priorities. Furthermore, the initial work was done rapidly (within one summer) and with very little money (mainly part-time salaries for students and interns during that summer). Most of these were engineering students (approximately 20 students) or medical students (14), in addition to one research student and one family medicine resident. Developing the products required very little equipment, and the programming was done by engineering students, so very little computer literacy was needed by the rest of the team. Scrum facilitated a unique process that afforded a high level of interdisciplinary collaboration.

Effective teamwork is paramount in all healthcare organizations, yet research of interventions and approaches to increase teamwork in these organizations has been narrow in scope, concentrated in hospital settings and on tools, programs, and organizational structures [23]. As such, there is a gap in understanding about methods to promote teamwork in interdisciplinary research-based project teams within healthcare organizations and learning environments. A flexible approach will be needed to support the emergence of innovative tools such as artificial intelligence (AI) in medical settings [24]. Due to its dynamic and collaborative approach, Scrum is increasingly cited as an effective method for different types of interdisciplinary research processes and scientific projects, such as bridging the gap between research and practice in academia-industry work [16, 18]. Scrum could be a useful way to structure multidisciplinary teams within healthcare organizations that would allow a dynamic response to the introduction of AI and other new technologies.

The incremental and collaborative approach to product development ensured that products were evaluated by a diverse set of stakeholders throughout the development process. Each step was reviewed by the entire project team during Sprint Reviews, allowing for knowledge users, content experts, and researchers to contribute a variety of feedback. This resulted in products being shaped in unexpected ways based on real-time experience and made it easier to identify gaps early. For example, the literature review team had very broadly defined objectives at the outset. The collaborative and incremental approach allowed the literature review team’s objectives to be directly informed on an ongoing basis by challenges faced by the development teams and narrowed/tailored to fit their needs. The iterative nature of Scrum allowed the team to develop new goals for each Sprint based on findings and discussions from the previous Sprint.

The process of developing digital learning tools with Scrum provided a distinctive learning opportunity for students. Scrum is increasingly seen as a priority skill for software development students, as it is a method often used in the field [25]. However, the method is not routinely taught to medical students. The Scrum approach facilitated students learning new skills, such as research and curriculum development. More significantly, students were taught how to work with an interdisciplinary team. Scrum methodology mandates that all elements of the project should be completed by the team. As such, team members from different disciplines exchanged ideas and supported each other to complete the projects. Because we involved the knowledge users (medical students and residents) directly in the design process, we were able to use their perspective and experience to create digital resources that would best meet their needs.

Though Scrum was highly effective, there were limitations to the method. Scrum dictates that each Sprint should be of the same duration – an outcome that is not always feasible during complex interdisciplinary projects. For example, research timelines tend to be very different from standard product development timelines. Processes like obtaining research ethics board approval required more time to complete than the duration of any individual Sprint. Scrum should be adapted when needed to accommodate the time constraints and limitations that arise from working within an interdisciplinary team [18].

The project was expanded in May of 2021 with the development of other digital learning tools identified by DFM members. Research is being carried out in parallel to ensure decision-making is evidence-based. We are continuing to expand and finalize the current modules and are developing additional tools (for example, an Indigenous Musculoskeletal Health module that will be developed by an Indigenous technology company and a series of Indigenous Health modules that will involve stakeholders from the university’s Indigenous Resource Centre, Indigenous Faculty members, and others. A Supporting Psychological Health in First Responders (SPHIR) grant was recently received from the Province of Alberta to expand the Scuba VR program for the management of post-traumatic stress injuries in first responders. Additionally, relevant knowledge about digital learning tools is being generated that can be shared with other institutions and translated into practice. Lessons learned from these early projects will help future teams to develop more complex digital learning tools. For example, colleagues plan to use AI and natural language processing to mine existing datasets of questions asked by healthcare providers to identify areas and domains where knowledge and practice can be enhanced through continuing medical education. This will require the formation of an expert interdisciplinary team. We are exploring additional opportunities with AI, such as whether a voice-to-text AI scribe application can be used to update medical records to minimize the administrative burden on family doctors, or whether AI could develop labour-intensive remediation and academic plans for our residents and faculty. Our learnings from the above digital learning tool development and application of the Agile Scrum Methodology will facilitate this work and that of other innovators.

## Conclusions

Agile Scrum Methodology allowed the five teams to complete their objectives, including the development of prototypes for three digital learning tools, a website to support dissemination, and a library of supporting literature and related terms, efficiently and at a low cost. The Scrum method was also effective for the development of additional digital learning tools after the initial Scrum. Scrum’s interdisciplinary and incremental approach allowed gaps to be identified and addressed in real time. Developing digital learning tools with the involvement of medical students and other knowledge users, using innovative approaches such as Agile Scrum Methodology, has the potential to profoundly impact medical education, practice, and patient care. Scrum demonstrates promise and should receive further consideration as a method for developing learning tools in medical education.

## Data Availability

The datasets used and/or analysed during the current study are available from the corresponding author on reasonable request.

## Abbreviations

AI: Artificial intelligence
DFM: Department of Family Medicine
QI: Quality improvement
VR: Virtual reality
